# Inhalable extracellular vesicles as cell-free therapeutics for chronic respiratory disease

**DOI:** 10.3389/fbioe.2026.1811444

**Published:** 2026-04-23

**Authors:** Pinjie Zhang, Tingting Luo, Zhu Song, Hongxiang He, Yili Yang, Junfeng Jiang, Li Zhang

**Affiliations:** 1 School of Gongli Hospital Medical Technology, University of Shanghai for Science and Technology, Shanghai, China; 2 Department of Histology and Embryology, College of Basic Medicine, Naval Medical University, Shanghai, China; 3 Department of Pathogen Biology, Naval Medical University, Shanghai, China; 4 Department of Pathology, Faculty of Medical Imaging, Naval Medical University, Shanghai, China

**Keywords:** cell-free therapy, chronic respiratory diseases, engineered modification, extracellular vesicles, inhalation delivery

## Abstract

Chronic respiratory diseases, such as chronic obstructive pulmonary disease (COPD), asthma and idiopathic pulmonary fibrosis (IPF), impose a significant burden on global health. The current drugs can mostly only alleviate symptoms or delay the progression, but are not very effective in reversing the structural remodeling, and are accompanied by obvious systemic adverse reactions. Extracellular vesicles (EVs), as nanoscale membrane-bound particles released by cells, possess excellent biocompatibility, low immunogenicity, and certain tissue targeting properties. They can also partially replicate the paracrine effects of cell therapy, providing a novel drug delivery platform for precise treatment of chronic respiratory diseases, and are particularly well-suited for inhalation administration. This review first provides an overview of the molecular profiles of major classes of native EVs, including those derived from mesenchymal stromal cells, pulmonary tissues, and non-pulmonary sources such as serum or plasma, platelets, and milk, and summarizes their respective therapeutic potentials in chronic respiratory pathologies. Subsequently, the key points focus on summarizing the research progress in engineering EVs through strategies such as optimizing cultivation conditions, surface targeted modification, and loading of active substances, in order to adapt them for inhalation delivery. Finally, from the perspectives of formulation and quality control, GMP scale-up, and regulatory pathways, the opportunities and challenges of realizing the integrated transformation of cell and gene therapy through engineered inhalable EVs in chronic respiratory diseases are discussed.

## 1 Introduction

Chronic respiratory diseases, such as COPD, asthma, and idiopathic pulmonary fibrosis (IPF), remain a major global public health burden (Wang et al., 2026; Jayasooriya et al., 2025; de Oca et al., 2025). These diseases share key pathological features, including chronic inflammation, persistent airway epithelial injury, pulmonary fibrosis, and airway remodeling (Purcell et al., 2025; Liu et al., 2021). Clinical complexity has been further compounded by persistent respiratory sequelae after COVID-19 (Singh et al., 2023; Cha et al., 2024). Current therapies can alleviate symptoms or delay progression to some extent, but fall short of enabling precise, long-lasting disease control. In COPD, available treatments mainly reduce symptoms and exacerbations rather than fundamentally modifying disease progression (Singh et al., 2025). In pulmonary fibrosis, pirfenidone and nintedanib cannot reverse established lung scarring and are often associated with adverse effects that result in poor patient tolerance (Li et al., 2022). These traditional drugs often have low bioavailability and easily get trapped by the lung’s natural barriers (Chai et al., 2025), thereby limiting their therapeutic potential. As such, developing new treatment strategies that are more precise, safe, effective, and suitable for long-term use has become a pressing priority in chronic respiratory disease research.

Extracellular vesicles (EVs), which are nano-sized membrane-bound particles secreted actively by cells, can carry various bioactive substances such as proteins, nucleic acids, and lipids. These molecules enable EVs to play crucial roles in processes including intercellular communication, inflammation regulation, immune response, and tissue repair (Jadamba et al., 2024). Notably, this type of natural carrier not only exhibits excellent biocompatibility and low immunogenicity but also demonstrates a certain degree of tissue tropism. And they can protect the loaded drugs from degradation in the body environment to some extent. Based on these characteristics, EVs provide an ideal carrier foundation for precise drug delivery in pulmonary diseases. Compared with traditional cell therapy and gene therapy, this cell-free therapy platform exhibits unique complementary advantages and holds broad development prospects (Sun et al., 2025; Li et al., 2025; Ridzuan et al., 2021). Against this backdrop, the combination of engineered EVs technology and the inhalation-based drug delivery method has paved a new way for treating chronic respiratory diseases. Inhaled administration can directly deliver EVs to the local lung lesions, significantly increasing the drug concentration in the lesion area and reducing systemic exposure and side effects. This highly matches the treatment needs of chronic respiratory diseases and makes it a treatment strategy with great clinical translation prospects (Gou et al., 2025; Anderson et al., 2022).

However, native EVs still have many inherent limitations in the clinical application of chronic respiratory diseases, which restricts their potential for clinical use. In recent years, interest in EV-based therapies has grown substantially, along with increasing attention to inhalable exosomes for pulmonary diseases. Most available reviews have emphasized either the general therapeutic roles of EVs or the promise of inhalation as a route of administration. By contrast, few reviews have focused specifically on the development of inhalable engineered EVs for chronic respiratory diseases. In this review, inhalable EVs are considered a cell-free therapeutic strategy for chronic respiratory diseases. We begin by summarizing the therapeutic potential and inherent limitations of native EVs. We then discuss the design principles and engineering strategies relevant to inhalable EVs, followed by formulation, aerosol delivery, and regulatory issues that may influence clinical translation. We also highlight shared mechanistic features across chronic disease progression to clarify where engineered inhalable EVs may have therapeutic relevance. Overall, this review seeks to provide a conceptual and methodological framework for the development and translation of EV-based inhalation therapies in chronic respiratory diseases.

## 2 Molecular characteristics and therapeutic potential of native extracellular vesicles

**FIGURE 1:**
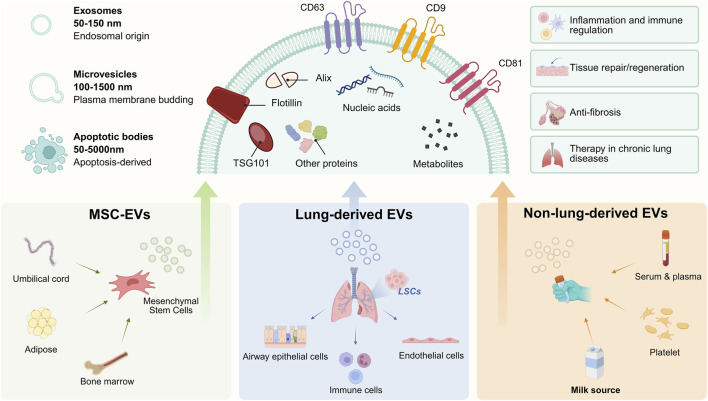
Native extracellular vesicle subtypes, sources, and therapeutic effects associated with chronic respiratory diseases. This figure illustrates characteristic membrane markers of EVs, such as CD9, CD63, CD81, and flotillin, along with their intraluminal components, including nucleic acids, metabolites, ALIX, and TSG101. Exosomes, microvesicles, and apoptotic bodies represent the primary EV subtypes. Major sources include MSC-derived EVs, pulmonary EVs, and non-pulmonary EVs. In chronic respiratory diseases, these native EVs exhibit multiple potential therapeutic advantages in inflammation and immune regulation, tissue repair and regeneration, and anti-fibrotic effects. Created by biorender.com.

EVs are nanometer-to-micrometer-sized membrane-bound particles released by cells. As a core medium for intercellular communication, their classification and functions exhibit high specificity. Based on common field classifications and related research, they can be primarily divided into three categories: endosome-derived exosomes (diameter 50–150 nm), microvesicles formed by budding from the plasma membrane (diameter 100–1,500 nm), and apoptotic bodies released during apoptosis (diameter 50–5,000 nm) (Greening et al., 2025; Welsh et al., 2024a). It is important to note that, according to the latest guidelines from the International Society for Extracellular Vesicles (MISEV2023), non-vesicular extracellular particles (NVEPs) are not classified as EVs and are therefore excluded from this discussion. Furthermore, due to overlapping sizes among EV subpopulations and the difficulty in rigorously proving their generation pathways, this review follows MISEV2023 recommendations and uses the generic term “EVs” throughout. Although different subtypes have differences, EVs generally contain a variety of bioactive molecules such as proteins, nucleic acids, and lipids. Their specific composition significantly depends on the type of source cells and their physiological states. And the tetraspanin protein superfamily (e.g., CD63, CD9, CD81) and endosome-related proteins (e.g., TSG101, ALIX) are frequently used as markers. They are essential for both the characterization and detection of these vesicles (Ripoll et al., 2026; Kalluri, 2024; Chen et al., 2024). The molecular composition of EVs gives them functional diversity, enabling them to play potential roles in processes like inflammation regulation and tissue repair. Due to their distinct characteristics, EVs from different sources have varying therapeutic potential and application scenarios in chronic respiratory diseases, collectively forming a diverse pool of candidate carriers. These inherent shortcomings also determine the necessity of targeted engineering modifications. The classification, main sources and functions are shown in Figure 1.

### 2.1 Mesenchymal stem cell-derived extracellular vesicles (MSC-EVs)

Mesenchymal stem cell-derived native extracellular vesicles (MSC-EVs) are isolated from conditioned media after culturing mesenchymal stem cells derived from umbilical cord, adipose tissue, and bone marrow (Kou et al., 2022). MSC-EVs retain the therapeutic potential of mesenchymal stem cells to a certain extent and can modulate pulmonary pathological processes through multiple mechanisms, making them one of the most widely used EV sources in chronic respiratory disease research (Zhu et al., 2023).

Regarding chronic inflammation regulation, MSC-EVs suppress the NF-κB signaling pathway, reduce expression of pro-inflammatory markers like IL-6 and TNF-α, and promote secretion of anti-inflammatory factors such as IL-10. Concurrently, they modulate macrophage function and drive polarization toward the M2 phenotype, thereby effectively alleviating pulmonary inflammatory responses (Li et al., 2025; Zhao et al., 2025). In terms of anti-remodeling and anti-fibrosis, MSC-EVs can downregulate the expression of TGF-β, α-SMA, Col1a1, and other fibrosis-related genes and proteins, while also reducing collagen deposition in lung tissue. They demonstrate potential for mitigating fibrosis in models of silicosis and idiopathic pulmonary fibrosis (Li et al., 2025; Zhao et al., 2025). Furthermore, MSC-EVs can regulate the STAT3/Krt8/AQP5 axis, promoting the transdifferentiation of type II alveolar epithelial cells into type I cells, which is beneficial for the repair of alveolar epithelium and enhancing the integrity of alveolar structure (Tang J et al., 2025).

Compared with MSCs, MSC-EVs also possess excellent biocompatibility and lower immunogenicity, indicating a significantly lower risk of immune rejection (Xu et al., 2020). Unmodified MSC-EVs typically carry tetraspanins CD9, CD63 and CD81, which are often used as markers of EV populations. Moreover, they can be taken up by lung epithelial cells such as MLE-12, BEAS-2B and A549, providing a basis for their exertion of local biological effects in lung tissues (Zhao et al., 2023; Xu et al., 2020).

### 2.2 Lung-derived extracellular vesicles (LDEVs)

Naturally present in the pulmonary microenvironment, lung-derived extracellular vesicles (LDEVs) are nano-to-microscale membrane vesicles released by epithelial, endothelial, and immune cells within the lungs and airways (Park et al., 2025).

LSC-Exo, a subtype of LDEV secreted by lung spheroid cells (LSC), possesses both lung-targeting properties and multiple therapeutic functions. It can retain the membrane localization and conformational characteristics of the naturally expressed ACE2 of LSC, and can bind to the RBD of the SARS-CoV-2 spike protein, achieving homologous lung targeting without genetic modification (Li et al., 2021; Wang Y et al., 2024). At the therapeutic mechanism level, LSC-Exo naturally carries anti-fibrotic miRNAs such as miR-30a-3p and miR-99a-5p, which can directly inhibit the TGF-β-mediated pro-fibrotic signaling pathway (Dinh et al., 2020). Compared to MSC-Exo, LSC-Exo demonstrates a superior targeting ability, enabling more precise delivery to the injured sites of the trachea, bronchioles, and pulmonary parenchyma, thereby enhancing localized therapeutic efficacy. Apart from LSC-Exo, other lung-derived EVs have also demonstrated potential in the diagnosis and treatment of chronic respiratory diseases (Wang Z et al., 2024). Bronchoalveolar lavage fluid-derived extracellular vesicles (BALF-EVs) carry proteins related to inflammation regulation and immunity, which can reflect the status of the pulmonary microenvironment and are suitable for the assessment of the severity of COPD (Fang et al., 2025). Human bronchial epithelial cell-derived EVs (HBEC-EVs) have been shown to mitigate pulmonary fibrosis by targeting TGF-β-WNT signaling crosstalk via key miRNAs such as miR-16 and miR-26a, both of which play regulatory roles in IPF pathogenesis (Kadota et al., 2021).

### 2.3 Non-lung-derived extracellular vesicles

Non-pulmonary native extracellular vesicles refer to a class of natural carriers derived from sources outside the lung. Representative examples include EVs originating from platelets, serum, human milk, and immune plasma. Although not directly sourced from the lung, these EVs represent valuable supplementary candidates for inhalation delivery, owing to their wide availability, ease of isolation, excellent biocompatibility, and potential for further engineering.

#### 2.3.1 Serum/plasma-derived extracellular vesicles

Serum or plasma, as one of the most clinically accessible sources of native EVs, can be obtained through routine blood collection. Soares Martins et al. demonstrated that using 250 μL serum or plasma as the initial sample, separated via commercial kits, and resuspending the final product in 200 μL PBS, resulted in serum exosome concentrations ranging from 5.3 × 10^8^ to 6.9 × 10^8^ particles/mL, while plasma concentrations reached 7.8 × 10^8^–9.9 × 10^8^ particles/mL (Soares Martins et al., 2018). Studies indicate that after intratracheal instillation of different doses of native mouse serum exosomes, no significant changes were observed in the proportion of immune cells or the expression of pro-inflammatory factors TNF-α, IL-1β, and IL-6 in bronchoalveolar lavage fluid (BALF) (Zhang et al., 2018). Clinically, nebulized convalescent human plasma-derived exosomes (ChipEXO™) have been used to treat severe COVID-19 pneumonia patients. Research reports indicate no clear allergic reactions or acute toxicity signals were observed in short-term follow-ups; however, the long-term safety of repeated administration and its extrapolation to chronic airway diseases requires further validation (Gül et al., 2022). Concurrently, native serum exosomes demonstrate exceptional precision targeting of pulmonary cells. Following intratracheal instillation or nebulized delivery, they exhibit relative enrichment in pulmonary macrophages (CD68^+^, F4/80^+^) (Zhang et al., 2018) and airway epithelial cells (pancytokeratin, Pan-CK^+^) (Han et al., 2022), effectively encapsulating small degradable nucleic acids such as siRNA and miRNA.

#### 2.3.2 Platelet-extracellular vesicles

Platelet-derived extracellular vesicles (PEVs) are nanoscale membrane vesicles secreted by platelets during activation, apoptosis, or storage. They can be isolated from platelet concentrates, platelet lysates, platelet-rich plasma (PRP), or expired platelet preparations. Clinical-grade PEVs can be prepared through standardized procedures, offering stable sources and ease of standardization (Fan et al., 2024; Ohayon-Steckel et al., 2026). As a natural carrier, PEVs exhibit superior biocompatibility compared to synthetic nanoparticles. In some studies, PEVs have shown good tolerance and no obvious toxic signals (Johnson et al., 2021). They remain functional after freeze–thaw cycles, addressing the limited shelf life of fresh platelets and supporting clinical storage and transport (Lopez et al., 2019). PEVs can also carry anti-inflammatory drugs including TPCA-1 and dexamethasone, and target them to highly inflamed lung tissues. By inhibiting inflammatory cell infiltration in the lungs, they significantly mitigate cytokine storms in acute pneumonia (Fan et al., 2024). Xuan et al. administered a clinically purified platelet-derived exosome product (PEP) via nebulization to cigarette smoke (CS)-induced emphysema mice, and found that PEP significantly alleviated CS-induced pulmonary emphysema in mice through multiple mechanisms. These included suppressing inflammation by downregulating the NF-κB signaling pathway, increasing the proportion of CD4^+^ FOXP3^+^ CTLA4^+^ regulatory T cells, and reducing the infiltration of S100A8/A9^+^ pro-inflammatory macrophages to modulate immune balance. PEP nebulization caused no toxic damage to normal lung tissue or organs such as the heart, liver, and kidneys in mice. It efficiently targeted the alveolar region and was specifically taken up by type I and type II alveolar epithelial cells and macrophages (Xuan et al., 2024).

#### 2.3.3 Milk-derived extracellular vesicles

Milk-derived extracellular vesicles (mEVs) are naturally occurring nanoscale lipid bilayer vesicles in mammalian milk. They can be efficiently isolated from large quantities of skim milk, fully meeting the demands of large-scale applications (Munagala et al., 2016). Milk-derived EVs show no significant cytotoxicity in lung-associated cell lines (A549, WI-26 VA4, MH-S) and, in rat inhalation studies, caused no observable organ damage or blood parameter changes after six daily doses (Ren et al., 2025). However, the safety of chronic, long-term repeated inhalation requires systematic evaluation. Their nanoscale size and lipid bilayer architecture favor mucosal diffusion. Together with their low immunogenicity and biocompatibility, these properties make mEVs particularly attractive for pulmonary drug delivery (Jiang et al., 2024; Santoro et al., 2025; Ran et al., 2024). Beyond their role as delivery vehicles, mEVs exhibit intrinsic anti-inflammatory and barrier-protective activities. Native mEVs have been shown to suppress LPS-induced IL-1β and IL-6 secretion (Ren et al., 2025), reverse Poly I:C-induced bronchial epithelial barrier disruption, and reduce excessive IL-6 and IL-8 production by over 90% (Karra et al., 2022).

### 2.4 Summary comparison of native EV sources

Native EVs from different sources exhibit distinct but complementary advantages for chronic respiratory disease applications. MSC-EVs are supported by the most extensive evidence for immunomodulation, anti-fibrotic activity, and tissue repair, making them a major therapeutic EV source. LDEVs, particularly those derived from lung-resident cells, offer superior pulmonary relevance and may provide more intrinsic targeting to injured airway or alveolar sites. By contrast, non-lung-derived EVs, including serum/plasma-, platelet-, and milk-derived EVs, are often more accessible and scalable, with advantages in biocompatibility, availability, and translational feasibility. Therefore, while native EVs provide an important biological foundation, their source-dependent differences also highlight the rationale for subsequent engineering strategies to improve delivery efficiency, therapeutic specificity, and manufacturability.

## 3 Inherent limitations of native extracellular vesicles

MSC-EVs, LDEVs, and other non-pulmonary EVs provide multiple candidate carriers for inhalation therapy in chronic respiratory diseases. However, despite these natural advantages, unmodified EVs still present important limitations for clinical translation and practical application.

First, the targeting capability of native EVs primarily relies on intrinsic molecules on the surface of donor cells, resulting in a lack of specific recognition of pathological sites in chronic respiratory diseases. For instance, the cellular or tissue targeting of native mEVs depends on intrinsic interactions of their surface membrane proteins, glycosylation, and lipid composition, lacking active targeting capabilities and are unable to specifically bind to lung lesion cells, such as inflammation-associated macrophages or damaged epithelial cells (Ren et al., 2025). While unmodified serum-derived small extracellular vesicles (sEVs) can accumulate in mouse lungs after nebulization delivery, their targeting relies solely on the native nanoscale properties of sEVs and the physiological lung environment, lacking active targeting for specific pulmonary diseases (Han et al., 2022).

Second, chronic respiratory diseases require sustained therapeutic effects through repeated long-term administration due to the chronic nature of disease progression and persistent airway injury. However, native EV yield is highly dependent on donor cell type and culture conditions. Furthermore, samples from different sources are prone to co-isolation with non-vesicular particles like protein complexes and lipoproteins during separation, leading to fluctuations in purity and composition (Sódar et al., 2016; Welsh et al., 2024b; Karimi et al., 2018). For instance, MSC-EV yield is influenced by cell passage number and culture environment. Studies indicate that primary placental mesenchymal stem cells (PC-MSCs) exhibit senescence after routine passage to the 10th generation, and EVs secreted by cells of different passages may exhibit batch-to-batch variability due to cellular state differences (Tong et al., 2025). Such variability undermines batch consistency, purity control, and dose standardization, which are especially problematic for therapies intended for repeated long-term administration.

Moreover, the functions of natural EVs depend on the active molecules they carry, but the types and quantities of these molecules are determined by the inherent characteristics of the source cells and tissues. For example, CD24, as a constitutive immune checkpoint, plays a crucial role in regulating the production of cytokines and chemokines by strictly controlling the NF-κB pathway (Grigoropoulos et al., 2024). miR-148a-3p inhibits the β-catenin signaling pathway by targeting Hsp90b1, thereby reducing collagen secretion by fibroblasts (Jiang et al., 2025). But the content of CD24 and miR-148a-3p in natural EVs is limited, making it difficult to achieve the effective concentration required for disease treatment. Therefore, although native EV cargo may have biological relevance, it is often insufficient for robust and predictable anti-inflammatory or antifibrotic therapy without engineering enhancement.

In addition, native EVs lack protective mechanisms to some extent. Upon entering the body, they are easily recognized and cleared by the immune system. Additionally, factors such as mucociliary clearance and fluid dilution prevent their long-term retention in the lungs (Ren et al., 2025). Atomization shear, as well as the stress at the gas-liquid interface, the adsorption of device materials, and the stresses caused by drying/rehydration, may all induce vesicle aggregation, damage to membrane integrity, or leakage of the load, resulting in differences in functional activity before and after inhalation (Susa et al., 2023; Mayor et al., 2021). These limitations collectively restrict the clinical application of natural EVs. Targeted engineering and formulation optimization are needed to improve targeting precision, functional consistency, and aerosol robustness.

## 4 Engineering and optimization of native extracellular vesicles for chronic respiratory diseases

### 4.1 Mechanistic convergence across chronic respiratory disease progression

Chronic respiratory diseases differ in etiology and clinical phenotype, but their progression often follows a relatively shared pathological cascade rather than fully divergent disease-specific pathways. In asthma, COPD, pulmonary fibrosis, and related disorders, chronic inflammatory stimuli often lead to epithelial or endothelial injury and barrier dysfunction, followed by immune dysregulation, abnormal macrophage polarization, fibroblast activation, extracellular matrix deposition, and progressive airway or alveolar remodeling (Carlier et al., 2021; Zhou et al., 2024). The relative contribution of each process differs among diseases, but during chronic lung injury, these events remain closely connected and often form a mutually reinforcing pathological network.

**FIGURE 2:**
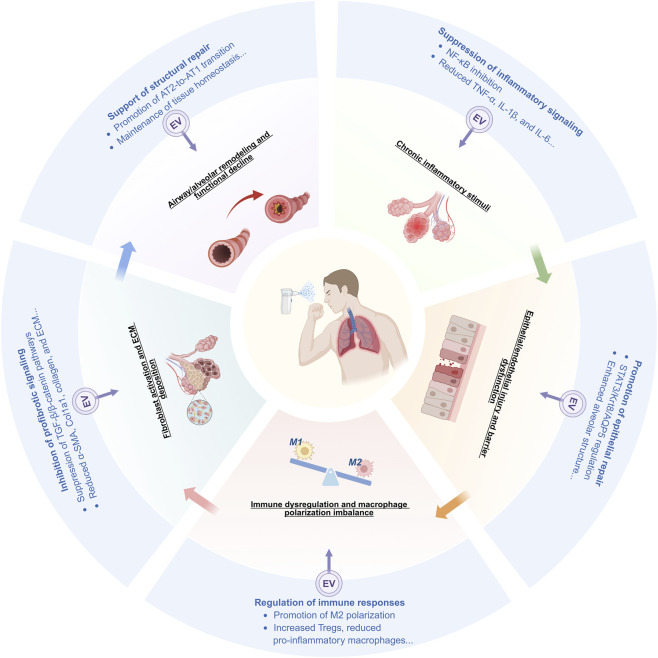
Mechanistic convergence across chronic respiratory diseases and key intervention nodes for engineered inhalable EVs. Chronic respiratory diseases with different etiologies and clinical features often converge on a shared pathological cascade. Native and engineered EVs may act at multiple points within this cascade. The mechanisms shown here are representative examples rather than an exhaustive summary. Created by biorender.com.

This convergence is therapeutically important because it points to a set of recurrent intervention targets across chronic respiratory diseases. Current evidence indicates that native and engineered EVs can modulate multiple steps in this cascade, including inhibition of inflammatory signaling, promotion of epithelial repair, regulation of macrophage polarization, attenuation of profibrotic pathways, and maintenance of tissue homeostasis (Coward-Smith et al., 2025; Liu et al., 2024; Zhao et al., 2025). From an engineering perspective, these shared mechanisms may also guide the selection of EV source, cargo design, targeting strategy, and formulation optimization (Cheng and Hill, 2022; Liu et al., 2023). Figure 2 outlines this integrative framework.

### 4.2 Design principles for inhalable engineered EVs

Chronic respiratory diseases are often characterized by the coexistence of persistent inflammation, epithelial injury, oxidative stress, immune dysregulation, and tissue remodeling (Katamesh et al., 2025). The design of inhalable engineered EVs should thus prioritize disease-relevant therapeutic goals over engineering efficiency alone (Cheng and Hill, 2022), to improve their biological relevance, local retention, and functional activity within the diseased lung microenvironment. Key considerations include selecting an appropriate EV source, matching the cargo or surface modification to disease-specific targets, and preserving vesicle integrity during formulation and nebulization (Tang Y et al., 2025; Liu et al., 2023).

Pulmonary administration also introduces additional constraints on EV stability and function (Han et al., 2022). Targeting modifications may enhance EV accumulation in inflamed or fibrotic regions of the lung, but their practical value depends on whether EVs maintain stability during formulation, nebulization, and deposition. Similarly, strategies that increase cargo loading should not compromise membrane integrity, cargo retention, or tolerability after repeated inhalation. The engineering of inhalable EVs should therefore strive for a rational balance between biological function, aerosol adaptability, and translational feasibility.

### 4.3 Optimization of donor cell culture conditions

The culture microenvironment of donor cells and their intrinsic physiological status directly dictate the quantity of EVs secreted, the profile of bioactive molecules loaded, and the resultant functional properties. Key influencing factors include the format of the culture system, oxygen tension, and the composition of the culture medium. Acidic pH can increase the secretion of EVs (Qu et al., 2023). Modifying the nutrient components in the culture medium, such as amino acids and glucose, may alter the biosynthesis and functions of EVs (Wang Y et al., 2024). External stimuli, such as inflammatory factor LPS, toxin CSE, and drug naringenin, can regulate the production and functions of EVs by changing the state of donor cells (Chen et al., 2022; Sun et al., 2024).

*In-vivo* microenvironmental cultivation mode can further enhance the anti-inflammatory and regenerative activities of EVs and improve their therapeutic adaptability (Xu et al., 2025; Kudinov et al., 2022). For instance, 3D droplet culture enables bone marrow mesenchymal stem cells (BMSCs) to produce EVs rich in bioactive molecules. These EVs contain osteogenic and angiogenic proteins as well as anti-inflammatory factors, a profile highly compatible with the therapeutic needs for anti-inflammatory and tissue repair in chronic respiratory diseases (Xu et al., 2026). Compared with traditional two-dimensional (2D) culture, 3D dynamic cultivation combined with ultracentrifugation increases exosome production by 20-fold, and further integration with tangential flow filtration (TFF) boosts yield by an additional 7-fold (Haraszti et al., 2018).

Hypoxic preconditioning represents another optimization strategy. Given the local hypoxic microenvironment characteristic of diseased lungs (e.g., in asthma), preconditioning MSCs with low oxygen enhances their self-renewal, proliferation, and EV secretion, thereby improving therapeutic effects (Dong et al., 2021). These hypoxia-conditioned EVs (Hypo-EVs) are better adapted to the target organ microenvironment. They are specifically enriched with anti-inflammatory and anti-fibrotic miR-146a-5p and epithelial barrier protection-related CAV-1, exerting targeted therapeutic effects by regulating signaling pathways such as STAT6 and TRAF6 (Xu et al., 2023; Luo et al., 2024).

### 4.4 Engineering of native extracellular vesicles

#### 4.4.1 Targeted modification techniques

Targeted modification involves introducing specific recognition molecules onto the surface of EVs or regulating their intrinsic targeting-related components through physical, chemical, or genetic engineering means (Gu et al., 2025). For chronic respiratory diseases, the core objective of targeted modification is to enhance EVs’ recognition capabilities toward pulmonary lesion microenvironments and target cells through endogenous or exogenous strategies, thereby improving local enrichment and cellular uptake efficiency for precision delivery.

Endogenous targeting modification involves genetically engineering donor cells to transfect exogenous plasmids encoding targeting molecules (e.g., peptides, antibodies, ligands). By leveraging cellular biosynthesis to stably deliver the targeted molecules to the surface of the EV membrane, this approach has the advantages of a relatively stable configuration and a lower risk of detachment (Song et al., 2022). The binding peptide (RBP) can specifically bind to the RAGE receptor, which is highly expressed on the surface of lung cells during inflammation. Kim et al. transfected HEK293 donor cells with an RBP-Lamp2b fusion protein plasmid to generate RBP-modified EVs (RBP-exo) (Kim et al., 2021). Compared with unmodified EVs, the enrichment of RBP-exo in pulmonary type I epithelial cells and macrophages was significantly increased. Beyond receptor-ligand recognition, leveraging the chemotactic signaling within the pulmonary microenvironment represents another strategy to enhance lung-targeted delivery. Wang et al. transfected the expression plasmid of the CXCR4 (C-X-C motif chemokine receptor 4) receptor into RAW 264.7 cells to obtain engineered EVs that presented CXCR4 on their surface (Wang et al., 2025). After being inhaled by nebulization, these EVs, through their specific binding to the ligands in the microenvironment of lung tissue via CXCR4, significantly enhanced the targeted delivery efficiency of the loaded drugs, successfully achieving the treatment of postoperative melanoma lung metastasis. This approach may extend to other pulmonary conditions requiring local enrichment, such as chronic inflammation or fibrosis.

Exogenous targeted modification involves anchoring the targeting molecules to the membrane surface through *in vitro* methods such as physical adsorption and chemical coupling after the EVs have been isolated and purified. It has the advantages of flexible operation and no need to modify the expression system of the donor cells (Hu et al., 2025). For airway inflammatory diseases, Tu et al. employed a strategy combining exogenous chemical coupling with RNA nanotechnology to attach pre-modified mannosylated (Man) PRNA-3WJ RNA nanoparticles to the surface of EV membranes through cholesterol-mediated anchoring, thereby targeting macrophage CD206 (Mrc1) (Tu et al., 2024). This enables the preferential uptake of pulmonary macrophages and the inhibition of Th2 inflammatory responses. Ren et al. utilized the high-affinity interaction between RGD peptides and integrin αvβ3 to prepare RGD-modified mEVs through the post-insertion method, aiming to target pulmonary inflammatory macrophages (Ren et al., 2025). Results demonstrated that in the lipopolysaccharide (LPS)-induced MH-S inflammatory macrophage model, RGD-modified mEVs significantly suppressed proinflammatory cytokines IL-1β and IL-6 more effectively than unmodified mEVs. This indicates that the RGD-mediated targeted binding can promote the internalization of mEVs by macrophages, increasing the effective concentration of local anti-inflammatory molecules.

#### 4.4.2 Active substance loading strategies

Native EV cargo is constrained by donor cell state, limiting consistent dosing and reproducible efficacy in chronic disease management. Loading exogenous agents or selectively enriching endogenous cargos offers a route to enhance EV potency and consistency. These strategies fall into two categories by loading stage: endogenous and exogenous loading.

Endogenous loading relies on transfection, co-culture, or drug pretreatment to introduce target molecules into donor cells for subsequent EV packaging. This strategy preserves EV membrane integrity and enables stable cargo loading (Nasiri Kenari et al., 2020). Studies have shown that EV-delivered miRNAs can directly modulate pulmonary inflammatory signaling, as exemplified by MenSC-EV-associated miR-671-5p through regulation of the AAK1/NF-κB axis (Lian et al., 2023). The endogenous miR-148a-3p in natural hucMSC-EVs can reduce fibroblast collagen secretion by targeting Hsp90b1, but its content is limited, and its anti-fibrotic efficacy requires enhancement. By constructing miR-148a-3p overexpressed hucMSCs, engineered hucMSC-EVs were obtained. These engineered hucMSC-EVs demonstrated a stronger ability to inhibit collagen deposition in the pulmonary fibrosis model (Jiang et al., 2025). In another strategy, lentiviral transduction enables 293T cells to express anti-CD206 scFv and CD63-L7Ae fusion protein. L7Ae binds the C/D box on mRNA, enabling efficient loading and enhanced delivery stability (Xiao et al., 2025).

Exogenous loading involves loading active substances into EVs after their separation and purification through methods including electroporation, sonication, incubation or chemical mediation (Guarro et al., 2025). This strategy is easy to operate and can be controlled in batches. It is suitable for different payloads such as nucleic acids, small molecules and proteins. Serum-derived sEVs can be loaded with cel-miR-39 mimics via electroporation for precise pulmonary delivery via nebulization. Chemically mediated methods like CaCl_2_ can enhance internalization and loading efficiency of siMyd88 through electrostatic interactions, enabling nebulized administration (Han et al., 2022). For small-molecule drugs, mild incubation enables passive loading while relatively preserving membrane integrity. Co-incubation of glycyrrhetinic acid (GA) with mEVs, for example, generates anti-inflammatory and anti-fibrotic effects in IPF (Ran et al., 2024).

Emerging loading technologies focus on enhancing loading efficiency and structural protection, supporting the scaled application of EV inhalation therapy. For instance, droplet extrusion microfluidics generates high shear forces through chip-based extrusion gaps, enabling efficient fusion of EVs and lipid nanoparticles (LNPs) within droplets (Chintapula et al., 2025). pH-dependent structural transitions in non-layered liquid crystalline lipid nanoparticles (LCNPs) enable spontaneous hybridization with EVs, improving nucleic acid loading compatibility (Bader et al., 2025). These strategies offer novel process options for scalable preparation, payload stability, and consistent efficacy in inhalation settings.

**FIGURE 3:**
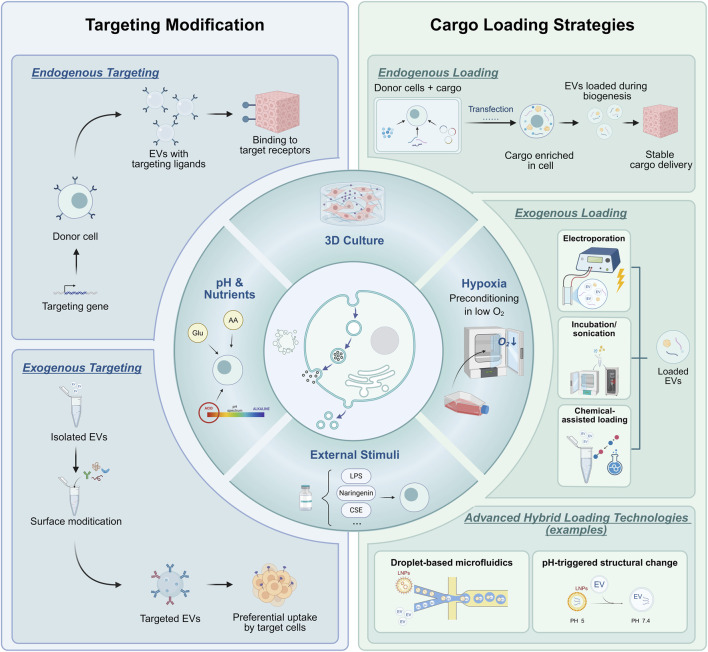
Engineering strategies of extracellular vesicles for the treatment of chronic respiratory diseases. This figure outlines the principal engineering strategies for customizing EVs tailored for chronic pulmonary disease therapy, encompassing three key aspects: optimization of donor cell culture conditions, surface-targeting modifications, and cargo loading. Created by biorender.com.

Together, these strategies, from culture optimization to targeted modification and cargo loading, constitute an engineered toolkit for inhalation therapy in chronic respiratory diseases. Figure 3 illustrates the overall framework.

## 5 Inhalation delivery adaptation and formulation optimization

The engineering strategies described above are primarily intended to improve targeting, therapeutic efficacy, and lesion-site specificity. However, modification approaches such as donor cell pretreatment, cargo loading, targeting technologies, and subsequent formulation-related processing may also alter the physicochemical properties of EVs. Furthermore, inhalation delivery is not simply a change in the route of administration. Aerosol generation, formulation state, storage conditions, and device-related stress may also influence vesicle integrity, dose consistency, and regional lung deposition. Accordingly, to successfully translate both native and engineered EVs into clinically viable inhalable therapeutic formulations, it is also necessary to take into account the advantages of the delivery route, formulation stability, and the quantitative bioengineering factors that determine aerosol performance.

### 5.1 Core advantages of inhalation delivery

Chronic respiratory diseases, including COPD, asthma, and IPF, primarily involve the airway epithelium, alveolar epithelium, and pulmonary interstitium. Shared features include persistent symptoms, airway remodeling, progressive lung function decline, and localized immune dysregulation, all of which require long-term intervention to slow disease progression (Liu et al., 2021; Purcell et al., 2025). A core therapeutic goal is therefore to achieve sustained drug action at pulmonary lesions while minimizing systemic exposure. Inhaled EVs from various sources and engineering strategies have shown anti-inflammatory, anti-fibrotic, and tissue-repairing effects in models of these diseases. Representative studies are listed in Table 1.

**TABLE 1:** Summary of key characteristics and therapeutic applications of inhalable engineered extracellular vesicles in chronic respiratory diseases.

Source of EVs	Delivery form	Chronic disease targeted	Engineering/Preconditioning strategy	Upregulation/Downregulation	Therapeutic effects	Reference
hUCMSCs	Nebulized inhalation	PF (including COPD-associated, IPF, ILD, post-inflammatory subtypes)	3D microcarrier culture	↑miR-486-5p, IL-10, HGF, M2 macrophage proportion ↓Col1a1, Col3a1, SPP1, MMP13, NF-κB	Alleviate pulmonary fibrosis, improve lung function, and are safe and effective	Li et al. (2025)
hUCMSCs	Nebulized inhalation	Chronic Asthma	Hypoxia-induced	↑ZO-1, E-cadherin, CAV-1 ↓p-STAT6, IL-4, IL-5, IL-13, Albumin concentration, total inflammatory cells, eosinophils, Goblet cell hyperplasia score, Collagen fiber percentage	Repair the airway barrier, inhibit asthma inflammation and remodeling	Luo et al. (2024)
hUCMSCs	Nebulized inhalation	Chronic Asthma	1. Hypoxia-induced 2. Genetic modification: Transfect exogenous miR-146a-5p mimics into Hypo-MSCs	↑Genes related to cell adhesion and cell junction, miR-146a-5p in lung tissue ↓ IL-4, IL-5, IL-13, OVA-specific IgE, collagen-1, α-SMA, TRAF6, TIRAP	Suppressing asthmatic inflammation and airway remodeling	Xu et al. (2023)
MSCs	Nebulized inhalation; Intratracheal installation (*i.t*.)	Asthma, COPD, IPF	1. Exogenous loading: loading small molecule drugs, miRNAs, and proteins through electroporation, incubation, ultrasound, freeze-thaw, and other methods 2.Endogenous loading: Regulate MSCs through endogenous regulation, LPS stimulation, hypoxia, or pretreatment with Toll-like receptor 3 agonists, so that the secreted EVs are enriched with miRNAs and proteins related to anti-inflammatory repair	↑IL-10, PGE2, TSG-6, KGF, HGF, VEGF, Ang-1, CD206, COPD: [EGF,FGF-2], IPF: [MMP-2 (tendency)] ↓TNF-α, IL-6, IL-1β, α-SMA, collagen deposition, Asthma: [IL-4, IL-5, IL-13, Allergen-specific IgE], COPD: [MUC5AC, MPO], IPF: [SMAD3, TGF-β, hydroxyproline deposition]	Exert anti-inflammatory, anti-fibrotic, and tissue repair effects by regulating the balance of cytokines, promoting M2 macrophage polarization, and enhancing alveolar epithelial/vascular repair	Fröhlich (2021)
skimmed bovine milk	Dry powder inhalation Cell co-incubation	Pulmonary inflammatory diseases	1. Surface modification: DSPE-PEG-cRGD was conjugated to the surface of mEVs via the post-insertion method to target integrin α_v_β_3_ 2. Carrier loading: RGD-mEVs were loaded into the borated cyclodextrin framework (BCF)	↓IL-1β,IL-6,TNF-α	Potently anti-inflammatory and efficiently released in response to the inflammatory microenvironment, with good biocompatibility	Ren et al. (2025)
skimmed bovine milk	Nebulized inhalation Cell co-incubation	IPF	Drug-loading modification: Loading glycyrrhetinic acid (GA) by co-incubation method	↓TGF-β1, Smad3, IL-6, IL-1β, TNF-α, Hydroxyproline	Anti-inflammatory and anti-fibrotic, improves IPF	(Ran et al., 2024)
platelet	Nebulized inhalation Cell co-incubation	COPD/emphysema	Product preparation: Reconstitution after lyophilization	↑BCL2L2, CTLA4, FOXP3, CD74 ↓TNF-α, IL-6, GM-CSF, CXCL5, CXCL12, NF-κB, JAK/STAT, Caspase1/3/8, HIF1αS100A8, S100A9, MMP9, CSF3R	Attenuates CS-induced COPD/emphysema by suppressing inflammation, apoptosis and oxidative stress, and modulating Treg cells	Xuan et al. (2024)
LSCs	Nebulized inhalation	IPF	Product preparation: Reconstitution after lyophilization	↑AQP5, ProSPC, vWF, MMP-2 (tendency) ↓α-SMA, Hydroxyproline, IL-4, MCP-1, Caspase-3, Cleaved PARP, Proportion of TUNEL-positive cells, SMAD3	Promotes alveolar/vascular repair, inhibits fibrosis, apoptosis and inflammation, improves lung function, and thereby attenuates bleomycin- and silica-induced pulmonary fibrosis	Dinh et al. (2020)
HEK293T	Intratracheal installation (*i.t*.)	Asthma (cockroach allergen-induced allergic airway inflammation)	1. Load modification: miR-511-3p mimics were loaded into EVs via ExoFect transfection combined with heat shock method 2. EVs are modified with PRNA-3WJ nanoparticles (containing mannose ligands) to target the macrophage Mrc1 (CD206) receptor	↑Arg-1, Chi3l3, Ym1/2 ↓IL-4, IL-5, IL-13, IL-1β, TNF-α, iNOS, C3, Cockroach allergen-specific IgE, IgG1	Targets lung macrophages via Mrc1/CD206, polarizes to M2, inhibits C3 and Th2 inflammation, and attenuates cockroach allergen-induced allergic airway inflammation in asthma	Tu et al. (2024)

Although intravenous infusion can achieve systemic exposure, it inevitably suffers from the preferential clearance and non-targeted distribution by the mononuclear phagocyte system in the liver and spleen (Aimaletdinov and Gomzikova, 2022). This results in limited effective exposure in the lungs and an increase in the dosage requirement, while also increasing the need for cold chain and medical support. For the oral route, it needs to pass through gastric acid, digestive enzymes and the intestinal barrier, resulting in greater uncertainty regarding systemic exposure. This route is more suitable for local targeting or immune regulation scenarios in the gastrointestinal tract. It is difficult to directly meet the core requirements of local high exposure in the lungs and long-term repeated administration (Mondal et al., 2023; Zhang et al., 2025).

Inhaled EVs can reduce systemic exposure and invasiveness while delivering a concentrated effective dose to lung target tissues. They also avoid the uncertainties caused by the digestive environment of the gastrointestinal tract and the first-pass effect on the vesicles, reducing dose wastage and meeting the local treatment needs of chronic respiratory diseases. From a safety standpoint, inhalable EVs achieve high local exposure in the lungs through local administration, while significantly reducing blood drug concentrations to minimize systemic toxicity and the risk of off-target effects. Preclinical studies report that repeated inhalation of MSC-EVs does not trigger pulmonary inflammation or immunogenicity, offering a favorable safety profile compared to intravenous injection (Fröhlich, 2021; Kudinov et al., 2022). Inhalation administration can be accomplished through portable nebulizers without the need for anesthesia or professional operation, allowing patients to self-administer the medication. This is particularly suitable for elderly individuals and children, who are prone to chronic respiratory diseases and may have limited mobility (Xu et al., 2025c; Jansen et al., 2025). Nebulized inhalation also avoids invasive procedures, reducing infection risk and better meeting the long-term treatment needs of chronic diseases (Fröhlich, 2021).

### 5.2 Stability optimization strategies for inhalable extracellular vesicles

#### 5.2.1 Protective agents and excipients

Despite their therapeutic potential, inhalable EVs face stability challenges. As lipid membrane vesicles, they are sensitive to temperature shifts, freeze-thaw cycles, and interfacial stress. During nebulization, shear stress, osmotic pressure changes, and long-term storage aggregation can cause membrane rupture and cargo leakage. Addressing these stability issues is therefore critical for clinical translation.

Excipients and protectants are essential for the development of stable inhalable EV formulations. Excipients are mainly used to optimize the characteristics of dosage forms. They enhance the dispersibility, fluidity and lung deposition efficiency of EVs to adapt to different inhalation administration methods, while also assisting in maintaining the dispersion stability of EVs and reducing particle agglomeration. Protectants focus on maintaining the structural and functional stability of EVs during the preparation, storage, atomization and *in vivo* delivery processes, thereby reducing the loss of active ingredients effectively extends the formulation’s shelf life. Both of them need to have high biocompatibility, be non-toxic and non-irritating, and minimally interfere with EV bioactivity and targeting properties.

Commonly used excipients and protectants can be roughly classified into four categories: sugars, amino acids, surfactants and proteins, buffer salts, and osmotic pressure regulators (Yousry et al., 2024). Among them, carbohydrates including sucrose, trehalose, mannitol and inulin are the most widely used. During freezing and drying, they protect vesicle membranes by displacing water and inducing vitrification, which reduces ice crystal formation, dehydration, and phase separation. They also serve as solid matrices that regulate dry powder particle structures (Yousry et al., 2024; Jansen et al., 2025). Inulin, for example, forms hydrogen bonds to maintain EV membrane integrity and inhibit aggregation during nebulization. After 12 weeks at −20 °C, Inulin-preserved EVs can retain the supportive effect of EVs on lung organoid formation. After freeze-drying, they can still maintain good particle size and regeneration ability when stored at 20 °C and 43% relative humidity for 12 weeks (Jansen et al., 2025).

Amino acids such as leucine act as anti-agglomerating agents. When co-spray-dried with inulin and 4% leucine, EVs formed a dry powder inhaler (DPI) formulation. Cascade impactor analysis showed a fine particle fraction (FPF) of 46.53% (<5 μm) and a mass median aerodynamic diameter (MMAD) of 3.6 μm, suggesting potential for deep lung deposition (Jansen et al., 2025).

#### 5.2.2 Formulation and storage for inhalation

In inhalation delivery settings, EVs typically undergo formulation steps like nebulization or dry powder conversion, followed by challenges in storage and transportation conditions. Formulation feasibility must therefore be considered in conjunction with engineering design requirements.

Liquid nebulization and dry powder inhalation are the two primary delivery platforms. Liquid nebulization is easier to operate and less dependent on inhalation flow rate, making it suitable for patients with limited lung function or those requiring flexible dosing. The key optimization challenge is minimizing inactivation during nebulization and controlling fluctuations in the effective delivered dose (Dhand et al., 2018). The delivery efficiency can be evaluated from three aspects: the output dose and recovery rate, the consistency of structure before and after atomization, and the maintenance of function before and after atomization. Jet nebulizers (JNs), vibrating mesh nebulizers (VMNs), and ultrasonic nebulizers (UNs) are the main devices used for liquid aerosol delivery. JNs are widely used and can accommodate liquid formulations, but repeated recirculation of the liquid during aerosol generation may subject EVs to greater mechanical stress and reduce delivery efficiency (Matthews et al., 2020; Danelli et al., 2021). VMNs typically produce more homogeneous aerosols with lower residual volume, although issues such as membrane clogging and formulation viscosity still need to be addressed (Galindo-Filho et al., 2019; Lin et al., 2020). UNs enable efficient aerosol output, but the heat generated during operation may be unfavorable for fragile vesicular systems (Niven et al., 1995). These differences suggest that device selection affects not only ease of use, but also EV integrity, cargo preservation, and dose reproducibility.

Dry powder formulations provide clear benefits in storage and usage convenience, lowering dependence on ultra-low temperature cold chains for chronic disease management. The key research focus is ensuring that EVs maintain structural and cargo stability during drying and rehydration, and that they rapidly reconstitute and retain functionality upon entering the lung’s liquid environment (Golan and Stice, 2024). Compared with liquid nebulization, dry powder inhalers offer better long-term storage potential and greater portability, but they impose stricter requirements on powder engineering, dispersibility, and rehydration stability after deposition in the respiratory tract (Shahin and Chablani, 2023; Omidian et al., 2025).

**FIGURE 4:**
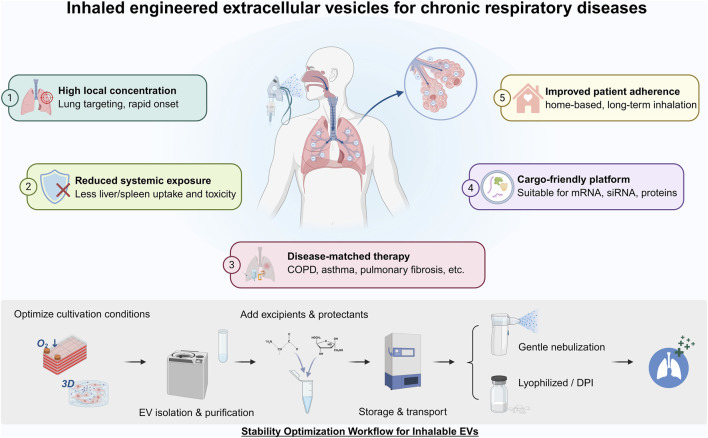
Advantages of inhaled extracellular vesicles in chronic respiratory disease treatment and stability optimization workflow. This figure demonstrates the multiple advantages of EVs delivered via inhalation in chronic respiratory diseases and briefly outlines the workflow for optimizing the stability of inhaled EV products. Created by biorender.com.

During storage and transportation, repeated freeze-thaw cycles can lead to a decrease in EV concentration and RNA content, an increase in aggregation, and diminish biological activity. Accordingly, improving both shelf-life and in-use stability represents a critical priority for EV formulation development (Ahmadian et al., 2024). Figure 4 outlines the core advantages of inhalable EVs and their preparation workflow, from cell culture to nebulization or dry powder administration.

### 5.3 Quantitative bioengineering considerations in EV aerosol delivery

For inhalable EV therapies, delivery outcomes are shaped not only by the intrinsic bioactivity of the vesicles, but also by the physical factors that control aerosol transport and deposition in the respiratory tract. EVs are nanoscale vesicles, yet their own diameter does not solely determine where they deposit after inhalation. What matters more during pulmonary delivery is the aerodynamic diameter of the droplet or particle in which the EVs are contained (Patton and Byron, 2007; Guo et al., 2021). Aerosols within the 1–5 μm range are more favorable for lower respiratory tract delivery. These particles can be retained in regions of reduced airflow through gravitational settling. Particles above 5 μm tend to deposit in the upper airway or central conducting airways because of inertial impaction. And particles below 1 μm are weakly influenced by gravity and inertia. They tend to remain suspended in the airflow, and are often exhaled before meaningful deposition occurs, which limits deep-lung delivery. The target deposition region within the lung also varies with the disease setting. Conducting-airway deposition may be preferable for disorders primarily affecting the airways, such as asthma, whereas diseases involving the small airways, alveoli, or interstitium are more likely to require efficient peripheral lung delivery (Karra et al., 2019; Usmani et al., 2005). *In vitro* aerodynamic testing, when combined with model-based estimation of regional deposition, may offer a more useful basis for matching formulations with inhalation devices and for anticipating translational performance (Walenga et al., 2025).

The nebulization process itself can also challenge EV stability. During aerosol generation, EVs may encounter shear forces, air–liquid interfacial stress, pressure fluctuations, and, in some devices, heat. Evidence from studies on nebulized proteins and other biologics indicates that these stresses are capable of damaging structural integrity. JNs atomize liquids by means of high-pressure gas, a process associated with considerable shear stress. Under such conditions, proteins including lactate dehydrogenase and urease have shown log-linear degradation, while IgG and G-CSF undergo a rapid loss of native conformation within 5–10 min, together with marked aggregate formation (Matthews et al., 2020). UNs raise a different set of concerns. Their piezoelectric mode of aerosol generation can introduce both thermal stress and cavitation-related shear. In the absence of cooling, the liquid temperature may rise to a level close to the unfolding temperature of many biologics; lactate dehydrogenase, for instance, has been reported to lose its enzymatic activity completely after 20 min of ultrasonic nebulization (Niven et al., 1995).

Device-dependent aerosolization mechanisms also produce clear differences in aerosol performance and drug output. One study in patients with moderate-to-severe stable COPD receiving nebulized bronchodilator therapy during noninvasive ventilation found that pulmonary delivery with a vibrating mesh nebulizer exceeded that of a jet nebulizer by more than threefold (Galindo-Filho et al., 2019). The vibrating mesh device achieved a higher inhaled dose (22.78% ± 3.38% vs. 12.51% ± 6.31%) and a higher lung deposition dose (12.05% ± 2.96% vs. 3.14% ± 1.71%). Its residual volume was substantially lower as well (3.08% ± 1.3% vs. 46.44% ± 5.83%, p = 0.001), and deposition was improved across lung regions. JNs still retain practical value because they are less expensive, structurally simple, and easy to use (Gardenhire et al., 2023).

These studies were not conducted with EV formulations, but they still point to issues that are highly relevant to EV aerosol delivery. Mechanical and thermal stresses generated during aerosolization are likely to be unfavorable for fragile vesicular systems, and nebulizer choice can alter emitted dose, residual volume, and regional lung deposition to a considerable extent. For this reason, evaluation of inhalable EV formulations should extend beyond vesicle quality alone. Aerosol performance metrics, such as mass median aerodynamic diameter (MMAD), fine particle fraction (FPF), emitted dose, and estimated regional deposition, should be reported to allow more meaningful comparison across studies (Jabbal et al., 2017). These parameters would also provide a more practical framework for formulation–device selection and future clinical translation.

## 6 Challenges and clinical translation

### 6.1 Long-term safety considerations for repeated pulmonary administration

For chronic respiratory diseases, inhalation offers unique practical advantages, such as localized delivery, the feasibility of repeated administration, and compatibility with outpatient or home care. Yet the safety issues caused by long-term repeated inhalation still need attention. Upon inhalation, EVs directly interact with the airway epithelium, alveolar surfaces, and resident immune cells. Such interactions may affect local immune homeostasis and potentially lead to activation, suppression, or other immunological abnormalities (Xia et al., 2024). While inhalation is more localized than intravenous routes, systemic exposure remains a concern. Inhaled substances may cross the alveolar-capillary barrier and enter the circulation, especially when barrier integrity is compromised by disease (Carlier et al., 2021; Jin et al., 2023). This complexity is further compounded in chronic respiratory conditions, where mucus hypersecretion, chronic inflammation, epithelial injury, and airway remodeling frequently alter aerosol deposition, retention, and clearance (Pangeni et al., 2023; Darquenne et al., 2024). Consequently, a fixed nominal dose does not necessarily ensure consistent tissue exposure over time, and repeated administration carries an increased risk of cumulative local or systemic effects.

For engineered EVs, immunogenicity requires specific vigilance. Surface modifications or targeting ligands can enhance delivery efficiency, but these alterations also change how vesicles are perceived by the immune system. Compared to native EVs, engineered variants introduce additional immunogenicity concerns (Xia et al., 2024; Liu et al., 2023). This is because their non-native surface features and altered membrane compositions may compromise pulmonary tolerance upon repeated administration. Beyond immunological effects, other safety aspects such as procoagulant activity and off-target biodistribution also warrant careful evaluation (Yang et al., 2024; Gupta et al., 2023).

Thus, the long-term safety assessment of inhalable engineered EVs should not be limited to short-term tolerance. Future studies need to prioritize repeated-dose inhalation models, evaluate chronic immunotoxicity, and assess the safety consequences of EV engineering modifications.

### 6.2 Current evidence and translational challenges

Available preclinical data suggest that engineered EVs could serve as viable inhaled therapies for chronic respiratory diseases by merging the innate biological functions of EVs with the benefits of targeted pulmonary delivery (Popowski et al., 2022; Liu et al., 2024). However, the current evidence base still has strong heterogeneity. Discrepancies in EV sources, engineering strategies, dosing regimens, and evaluation endpoints impede direct comparisons of efficacy and safety, thereby complicating translational assessments (Mizenko et al., 2024). Most findings remain confined to preclinical stages, with limited clinical progress. Many issues related to long-term treatment, including dosage selection, safety of repeated use, device compatibility, and patient usage scenarios, have not yet been fully resolved.

The lack of standardized potency and quality control systems across studies further obstructs clinical translation. The MISEV2023 guidelines recommend that EV research should systematically characterize physical properties, identity and purity, along with functional activity, thereby laying the foundational technical groundwork for the establishment of Critical Quality Attributes (CQAs) (Welsh et al., 2024a; Giebel and Lim, 2025). Parameters specific to inhaled delivery, such as aerosol output and aerodynamic particle size, should also be incorporated as critical quality attributes to meet FDA requirements for CMC documentation. Potency assays must prioritize core functions like immunomodulation and tissue repair, and should rely on multidimensional, quantifiable systems instead of single readouts to better support regulatory filing and batch consistency.

Methods that are feasible on a laboratory scale often face challenges in industrial-scale production. Typical hurdles include low yields, significant batch-to-batch variability, structural damage during purification, and a heightened quality control burden. The primary bottleneck is not a single technology itself, but rather how to scale up upstream production while maintaining batch consistency and limiting damage during downstream processing. Enhanced process standardization and automation may mitigate the impact of physical and chemical stresses during manufacturing.

These issues collectively continue to constrain the clinical translation of inhalable engineered EVs.

### 6.3 Regulatory challenges for inhalable engineered EVs

Regulatory translation of inhalable engineered EVs remains difficult, largely due to the limited availability of detailed guidance for this product class. Current frameworks lack a fully defined pathway for EV therapeutics, especially when engineering modifications are paired with inhalation delivery (Verma and Arora, 2025; Takakura et al., 2024). This ambiguity creates uncertainty regarding product classification, quality expectations, and evaluation standards.

Integrating EV formulations with specific delivery devices further complicates the regulatory landscape. This added complexity stems from the dual nature of the product, which blends the biological variability of EVs with the stringent performance requirements of inhalation systems. Consequently, the product may need to be regarded not only as a biological product or an EV-based therapy, but also as part of a drug-device combination system. Along with adherence to cGMP and CMC requirements, manufacturers must verify device performance, characterize aerosols, and validate the system under intended use scenarios.

Another challenge lies in terminology and reporting consistency. Using the terms “extracellular vesicles” or “exosomes” consistently and following MISEV2023 nomenclature can help reduce ambiguity. However, these consensus standards do not replace formal regulatory criteria. More granular guidance is essential for engineered EVs, particularly regarding source materials, surface modifications, potency testing, and inhalation-specific quality attributes (Pharmaceuticals and Medical Devices Agency, 2023; Welsh et al., 2024b).

## 7 Conclusions and outlook

COPD, asthma, and IPF share a chronic course, complex pathology, and progressive tissue remodeling. Traditional systemic or inhaled therapies rarely achieve all three of efficacy, safety, and long-term adherence at once. As an emerging biological delivery platform, EVs offer multitargeted, network-level effects on inflammation, immune balance, and tissue repair. Their native membrane structure, versatile loading capacity, and relatively low immunogenicity make them particularly suitable for inhalation delivery, allowing high local pulmonary exposure and long-term management.

Naturally occurring EVs from different sources display distinct anti-inflammatory, anti-fibrotic, and barrier-protective profiles, which provides a rich raw material pool for developing inhalable therapies. However, challenges persist for long-term application in chronic disease settings, including low targeting efficiency, insufficient yield and batch consistency, limited endogenous cargo, and poor airway retention and stability.

Strategies to overcome these limitations include modulating donor cell culture conditions (e.g., 3D or hypoxic culture) to enhance EV yield and activity, as well as using genetic or chemical modification to improve targeting of inflammatory macrophages, damaged epithelial cells, and fibrotic foci. Both endogenous and exogenous loading strategies can provide feasible routes for the stable and efficient delivery of diverse active substances, including nucleic acids, small-molecule drugs, and proteins. Integration with nebulization and dry powder technologies further positions inhalable engineered EVs as a promising platform that balances efficacy, convenience, and compliance.

However, achieving clinical translation requires systematic research across multiple dimensions. The safety of repeated long-term inhalation must be evaluated in chronic disease models, focusing on local immune homeostasis, systemic exposure, and cumulative risk. A quality framework aligned with MISEV2023 guidelines and inhalation CMC guidance should define CQAs, including physical attributes, identity/purity, functional potency, and aerosol/aerodynamic performance, to enable comparability across batches and manufacturing processes. Scalable manufacturing processes must minimize shear-induced damage while controlling costs. Regulatory classification and nomenclature for engineered inhalable EVs also remain unsettled, with possible regional differences in designation as biologicals, gene/cell therapy products, or combination products. Extending the MISEV2023 framework to incorporate device characteristics could facilitate more structured communication between academia and regulators.

Overall, inhaled engineered EVs offer a treatment approach for chronic respiratory diseases that is both scientifically innovative and clinically feasible.
